# Systemic chemotherapy for treatment of advanced small bowel adenocarcinoma with prognostic factor analysis: retrospective study

**DOI:** 10.1186/1471-2407-11-205

**Published:** 2011-05-27

**Authors:** Dong Hoe Koo, Sung-Cheol Yun, Yong Sang Hong, Min-Hee Ryu, Jae-Lyun Lee, Heung-Moon Chang, Baek-Yeol Ryoo, Yoon-Koo Kang, Tae Won Kim

**Affiliations:** 1 Department of Oncology, University of Ulsan College of Medicine, Asan Medical Center, Seoul, Korea; 2 Department of Biostatistics and Clinical Epidemiology, University of Ulsan College of Medicine, Asan Medical Center, Seoul, Korea

**Keywords:** small intestine, adenocarcinoma, chemotherapy, propensity score

## Abstract

**Background:**

We sought to evaluate prognostic factors affecting overall survival (OS), and to investigate the role of palliative chemotherapy using propensity score-based weighting, in patients with advanced small bowel adenocarcinoma (SBA).

**Methods:**

Data from a total of 91 patients diagnosed with advanced SBA at the Asan Medical Center between January 1989 and December 2009 were retrospectively analyzed. Patients were split into two groups, those who did and did not receive palliative chemotherapy.

**Results:**

Overall, 81 patients (89.0%) died, at a median survival time of 6.6 months (95% confidence interval [CI], 5.5 - 7.5 months). The 40 patients receiving chemotherapy showed overall response and disease control rates of 11.1% and 37.0%, respectively, with OS and progression-free survival (PFS) of 11.8 months (95% CI, 4.6 - 19.0 months) and 5.7 months (95% CI, 3.5 - 8.0 months), respectively. The 41 patients who did not receive chemotherapy had an OS of 4.1 months (95% CI, 3.1 - 5.1 months) and a PFS of 1.3 months (95% CI, 0.8 - 1.7 months). Multivariate analysis showed that lack of tumor resection, non-prescription of chemotherapy, liver metastasis, and intra-abdominal lymph node metastasis, were all independently associated with poor survival outcomes. After inverse probability of treatment weighting (IPTW) adjustment, the group that did not receive chemotherapy was at a significantly higher risk of mortality (HR 3.44, 95% CI 2.03 - 5.83, p < 0.001) than were patients receiving chemotherapy.

**Conclusion:**

Palliative chemotherapy may improve survival outcomes in patients with advanced SBA.

## Background

Small bowel cancer is very rare, accounting for 0.46% of all malignancies in the United States and 0.35% in South Korea [1,2]. Adenocarcinoma of the small bowel (SBA) is the most common histological type of such cancer, constituting 35.8% of small bowel malignancies [3]. Although patients with SBA have poor prognosis, few reliable data are available because of the rarity of such tumors. The treatment of choice for SBA is curative surgical resection. However, no standard protocol has been defined for use when SBA relapses or is unresectable because of locally advanced or metastatic status. Although several retrospective analyses have found that chemotherapy offers survival benefits in such patients, no prospective study has compared palliative chemotherapy with supportive care [4].

Propensity score-based weighting is a rigorous statistical technique permitting nonrandomized comparisons, and theoretically permits all data from two groups of patients to be used [5]. We employed this method to adjust for selection differences between patients with advanced BSA who received either palliative chemotherapy or supportive care, and we compared survival outcomes in two groups adjusted in such a manner. Thus, propensity score-based weighting allowed us to evaluate prognostic factors affecting overall survival (OS) in patients with advanced SBA, and to explore the role played by palliative chemotherapy in treatment of the disease.

## Materials & methods

We searched the Asan Medical Center Cancer Database to identify all patients who had been diagnosed with SBA at the Asan Medical Center (Seoul, Korea) between January 1989 and December 2009. Patients were included if they were ≥ 18 years of age; had histologically confirmed SBA with documentation of locally advanced, recurrent, or metastatic disease; and had no history of other malignancies. Patients with cancer of the ampulla of Vater or periampullary cancer were excluded. Of the 238 patients screened, 91 fulfilled all inclusion criteria. Patients with ampulla of Vater and peri-ampullary cancer (110 patients) and initially resectable disease (37) were excluded. Patients were divided into two groups, those who did or did not receive palliative chemotherapy. Patient medical records were reviewed to extract demographic data, tumor characteristics, type of treatment, response to treatment, and survival information. The protocol of this retrospective study was approved by the Institutional Review Board of the Asan Medical Center.

The primary endpoint of the study was OS. Secondary endpoints in the chemotherapy group were progression-free survival (PFS) and response rate (RR). OS was measured from the date of diagnosed advanced SBA, or confirmed recurrence, to the day of death from any cause, or was censored at last follow-up. PFS was measured from the date on which chemotherapy commenced to the day on which tumor progression or death from any cause was noted, or was censored at last follow-up. Survival curves were constructed using the Kaplan-Meier method and compared using the log-rank approach. Multivariate analysis defining factors associated with survival utilized a stepwise Cox's proportional hazard regression model. Differences in baseline characteristics between patients who did and did not receive systemic chemotherapy were compared using the *t*-test or the Mann-Whitney test for continuous variables, and the χ2 test or Fisher's exact test for categorical variables, as appropriate. To reduce the impact of treatment selection bias and potential confounding in this observational study, we used weighted Cox's proportional hazards regression models to adjust for significant differences in patient characteristics, using inverse probability of treatment weighting (IPTW) and robust standard errors [6]. Weights for patients receiving chemotherapy were the inverse of the [1-propensity score] values, and the weight for each patient not receiving chemotherapy was the inverse of the propensity score. All propensity scores were estimated without regard to outcomes, using multiple logistic regression analysis. A full model included the following factors: age, gender, primary tumor site, tumor histology, initial status, previous tumor resection, number of metastasis sites, liver metastasis status, peritoneal metastasis status, presence or absence of intra-abdominal lymph node (IALN) metastasis, lung metastasis status, presence or absence of bone metastasis, and Eastern Cooperative Oncology Group (ECOG) performance status (PS). Model discrimination was assessed using c-statistics (c = 0.786), and model calibration was evaluated employing the Hosmer-Lemeshow goodness-of-fit test (χ2 = 2.282, df = 8, p = 0.9711). A two-sided p value < 0.05 was considered significant, and 95% confidence intervals (CIs) were also calculated. All statistical analyses were performed using SPSS software (SPSS Inc., Chicago, IL).

## Results

**Figure 1:**
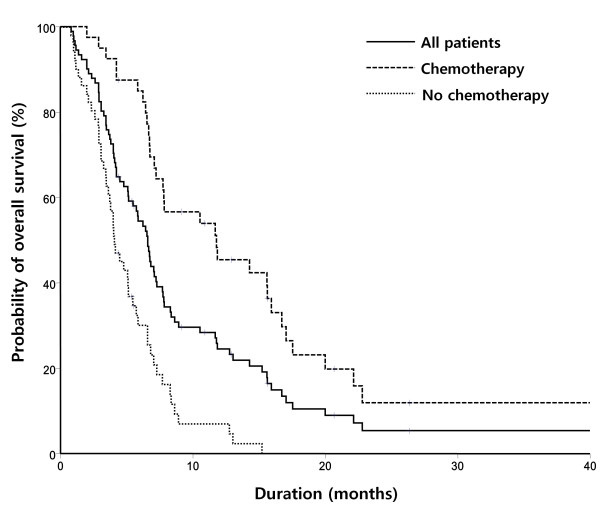
Overall survival (OS) curves of all patients and of those receiving or not receiving chemotherapy (CTx)

A total of 91 patients, of median age 58 years (range, 25-82 years), were followed-up for a median time of 10.0 months (range, 4.4 - 26.4 months). Overall, 81 patients (89.0%) died, with a median survival time of 6.6 months (95% CI, 5.5 - 7.5 months; Figure 1). At the time of diagnosis, 54 patients (59.3%) had ECOG PS of 0 to 1. A comparison of baseline characteristics is shown in Table 1. Patients in the chemotherapy group were significantly younger, and a significantly higher percentage had undergone resection of primary tumors. In contrast, higher proportions of patients in the non-chemotherapy group had IALN metastasis and poor PS. Of the 22 patients in the non-chemotherapy group who had good PS (0-1), 9 (40.0%) underwent bypass surgery, 2 (10.0%) palliative resection, and 1 (5.0%) stent insertion, whereas 10 (45.0%) received no treatment. Twenty of the 22 patients (91%) with good PS did not receive chemotherapy because of patient refusal, whereas 1 (5.0%) patient could not be treated because of the presence of pancreatitis, and 1 (5.0%) further patient did not receive chemotherapy because post-operative radiotherapy was prescribed.

**Table 1:** Pretreatment patient characteristics

		All patients	Palliative Chemotherapy	No Chemotherapy	p value
		n = 91	n = 40	n = 51	
Age (years)	Median (range)	58 (25 - 82)	55 (25 - 74)	59 (34 - 82)	0.03
Gender	Male	67 (73.6%)	31 (77.5%)	36 (70.6%)	0.46
ECOG PS	0/1	54 (59.3%)	32 (80.0%)	22 (43.1%)	<0.01
	2/3	33 (36.3%)	8 (20.0%)	25 (49.0%)	
	NA	4 (4.4%)	0 (0.0%)	4 (7.8%)	
Primary cancer site	Duodenum	71 (78.0%)	28 (70.0%)	43 (84.3%)	0.10
	Jejunoileum	20 (22.0%)	12 (30.0%)	8 (15.7%)	
Histology	WD/MD	44 (48.4%)	18 (45.0%)	26 (51.0%)	0.57
	Undifferentiated	38 (41.8%)	19 (47.5%)	19 (37.3%)	
	Undetermined	9 (9.8%)	3 (7.5%)	6 (11.8%)	
Status	Recurrent	17 (18.7%)	11 (27.5%)	6 (11.8%)	0.15
	Locally advanced	4 (4.4%)	2 (5.0%)	2 (3.9%)	
	Metastatic	70 (76.9%)	27 (67.5%)	43 (84.3%)	
Primary tumor	Resected	29 (31.9%)	19 (47.5%)	10 (19.6%)	<0.01
	Still present	62 (68.1%)	21 (52.5%)	41 (80.4%)	
Number of metastasis	0	4 (4.4%)	2 (5.0%)	2 (3.9%)	0.62
	1	50 (54.9%)	24 (60.0%)	26 (51.0%)	
	≥2	37 (40.7%)	14 (35.0%)	23 (45.1%)	
Liver metastasis	Yes	39 (42.9%)	14 (35.0%)	25 (49.0%)	0.18
Peritoneal metastasis	Yes	42 (46.2%)	19 (47.5%)	23 (45.1%)	0.82
IALN metastasis	Yes	41 (45.1%)	13 (32.5%)	28 (54.9%)	0.03
Lung metastasis	Yes	6 (6.6%)	2 (5.0%)	4 (7.8%)	0.59
Bone metastasis	Yes	3 (3.3%)	2 (5.0%)	1 (2.0%)	0.42
Treatment interval	1989-1999	25 (27.5%)	10 (25.0%)	15 (29.4%)	0.64
	2000-2009	66 (72.5%)	30 (75.0%)	36 (70.6%)	

Abbreviations: ECOG PS, Eastern Cooperative Oncology Group Performance Status; NA, not available; WD, well-differentiated; MD, moderately differentiated; IALN, intra-abdominal lymph node.

**Figure 2:**
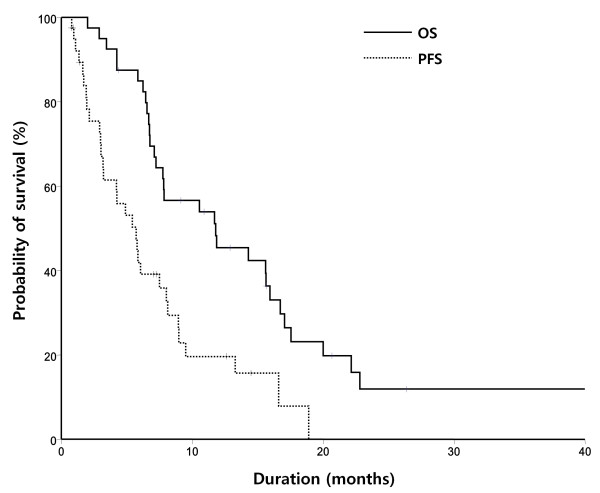
Overall survival (OS) and progression-free survival (PFS) curves of patients receiving chemotherapy

The chemotherapy regimens were based on fluoropyrimidine. Of the 40 patients who received chemotherapy, 25 (62.5%) were prescribed fluoropyrimidine and platinum, including 12 who received standard FP and 13 who took capecitabine-plus-cisplatin. Seven patients (17.5%) received oral fluoropyrimidine; three (7.5%) 5-FU plus leucovorin; and three (7.5%) fluoropyrimidine and irinotecan (FOLFIRI), whereas two (5.0%) received fluoropyrimidine, adriamycin, and mitomycin. The median number of chemotherapy cycles was 4 (range, 1-26 cycles). The overall response rate for the 27 patients with measurable lesions was 11.1%, with 3 patients showing partial responses, and the disease control rate was 37.0%, with 7 patients showing stable disease. OS and PFS were 11.8 months (95% CI, 4.6 - 19.0 months) and 5.7 months (95% CI, 3.5 - 8.0 months), respectively (Figure 2). In contrast, the OS and PFS of patients not receiving chemotherapy were 4.1 months (95% CI, 3.1 - 5.1 months) and 1.3 months (95% CI, 0.8 - 1.7 months), respectively.

In univariate analysis, the following factors were significantly associated with poor survival outcome: ECOG PS ≥ 2, duodenum as the primary tumor site, no previous tumor resection, liver metastasis, peritoneal metastasis, IALN metastasis, and absence of chemotherapy (Table 2). Upon subsequent multivariate analysis, four factors were independently associated with poor survival outcome: absence of tumor resection, liver metastasis, IALN metastasis, and absence of chemotherapy (Table 3).

**Table 2:** Univariate analysis of factors affecting survival.

Factor	Category	Event	Median OS	95% CI	p value
Age (years)	<58	44	6.7 m	6.0 - 7.4	0.41
	≥58	47	6.2 m	3.2 - 9.3	
Gender	Male	67	6.6 m	5.7 - 7.5	0.83
	Female	24	6.4 m	2.5 - 10.4	
ECOG PS	0/1	54	7.2 m	6.0 - 8.4	<0.01
	2/3	33	4.5 m	3.3 - 5.7	
	NA	4	2.6 m	0.0 - 6.9	
Primary site	Duodenum	71	6.2 m	5.0 - 7.5	<0.01
	Jejunoileum	20	11.9 m	5.1 - 18.6	
Histology	WD/MD	44	6.8 m	6.2 - 7.3	0.45
	Undifferentiated	38	5.1 m	2.3 - 7.9	
	Undetermined	9	8.3 m	0.1 - 16.6	
Initial status	Recurrent	17	10.5 m	4.0 - 17.1	0.21
	Locally advanced	4	6.8 m	5.9 - 7.8	
	Metastatic	70	5.8 m	4.3 - 7.4	
Primary tumor	Resected	29	11.8 m	3.8 - 19.8	<0.01
	Still present	62	5.2 m	3.5 - 6.8	
Number of metastasis	0	4	6.8 m	5.9 - 7.8	0.20
	1	50	7.1 m	5.8 - 8.4	
	≥2	37	5.5 m	2.7 - 8.3	
Liver metastasis	Yes	39	4.2 m	3.6 - 4.8	<0.01
	No	52	7.8 m	6.3 - 9.3	
Peritoneal metastasis	Yes	42	5.9 m	3.7 - 8.0	0.03
	No	49	8.6 m	5.5 - 11.8	
IALN metastasis	Yes	41	5.5 m	3.6 - 7.4	<0.01
	No	50	7.7 m	6.8 - 8.6	
Lung metastasis	Yes	6	3.1 m	0.0 - 6.9	0.14
	No	85	6.7 m	5.6 - 7.8	
Bone metastasis	Yes	3	5.2 m	2.4 - 7.9	0.75
	No	88	6.6 m	5.7 - 7.5	
Chemotherapy	(+)	40	11.8 m	4.6 - 19.0	<0.01
	(-)	51	4.1 m	3.1 - 5.1	

Abbreviations: ECOG PS, Eastern Cooperative Oncology Group Performance Status; NA, not available; WD, well-differentiated; MD, moderately differentiated; IALN, intra-abdominal lymph node.

**Table 3:** Multivariate analysis of factors affecting survival.

		HR	95% CI	p value
Chemotherapy	Not given	3.34	1.76 - 5.60	<0.001
Liver metastasis	Yes	2.43	1.51 - 3.91	<0.001
Primary tumor	Not resected	2.44	1.30 - 4.57	0.006
IALN metastasis	Yes	1.65	1.02 - 2.67	0.042
Primary site	Duodenum	1.19	0.55 - 2.56	0.986
Peritoneal metastasis	Yes	1.49	0.91 - 2.46	0.116
ECOG PS	2/3	1.03	0.61 - 1.75	0.807

Abbreviations: ECOG PS, Eastern Cooperative Oncology Group Performance Status; IALN, intra-abdominal lymph node.

**Figure 3:**
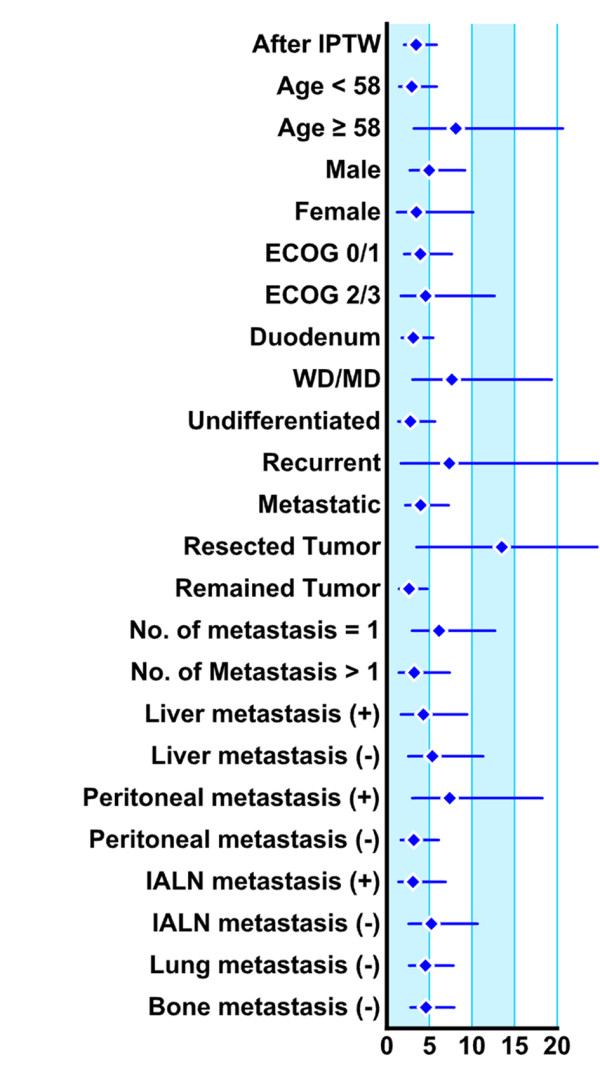
The effect of chemotherapy on subset analyses of overall survival (OS), with hazard ratios and 95% CI values for OS

Because the factor "no chemotherapy" showed the highest hazard ratio upon multivariate analysis (HR = 3.34), we evaluated the effect of chemotherapy on OS after adjusting for differences in patient characteristics between those in the chemotherapy and non-chemotherapy groups (please see Methods). After IPTW adjustment, non-chemotherapy group patients were at a significantly higher risk of mortality (HR 3.44, 95% CI 2.03 - 5.83, p < 0.001) than were those in the chemotherapy group. Further, of all factors analyzed, the absence of chemotherapy significantly affected OS, consistent with the data of the IPTW model (HR range, 2.61 - 13.49; Table 4; Figure 3).

**Table 4:** Effects of chemotherapy on overall survival (OS) of various patient subsets.

	Factors	N	HR	95% CI	p value
After IPTW	All factors	91	3.44	2.03 - 5.83	<0.001
Age (years)	<58	44	2.93	1.46 - 5.85	0.002
	≥58	47	8.10	3.17 - 20.67	<0.001
Gender	Male	67	4.97	2.69 - 9.18	<0.001
	Female	24	3.47	1.19 - 10.13	0.023
ECOG PS	0/1	54	3.93	2.03 - 7.63	<0.001
	≥ 2	33	4.55	1.64 - 12.65	0.004
Primary site	Duodenum	71	3.10	1.76 - 5.44	<0.001
	Jejunoileum*	20	NA		0.122
Histology	WD/MD	44	7.63	3.01 - 19.36	<0.001
	Undifferentiated	38	2.76	1.35 - 5.66	0.006
Status	Recurrent	17	7.33	1.64 - 32.75	0.009
	Metastatic	70	3.97	2.17 - 7.27	<0.001
Primary tumor	Resected	29	13.49	3.48 - 52.36	<0.001
	Still present	62	2.61	1.44 - 4.75	0.002
No. of Metastases	1	50	6.13	2.96 - 12.71	<0.001
	>1	37	3.21	1.40 - 7.38	0.006
Liver metastasis	Yes	39	4.29	1.65 - 9.43	<0.001
	No	52	5.34	2.52 - 11.31	<0.001
Peritoneal metastasis	Yes	42	7.37	2.98 - 18.27	<0.001
	No	49	3.16	1.64 - 6.09	0.001
IALN metastasis	Yes	41	3.06	1.36 - 6.89	0.007
	No	50	5.22	2.56 - 10.65	<0.001
Lung metastasis	Yes	6	NA		0.486
	No	85	4.53	2.62 - 7.82	<0.001
Bone metastasis	Yes	3	NA		0.809
	No	88	4.6	2.78 - 7.92	<0.001

* Deaths: 8/8 in the patient group not receiving chemotherapy; 7/12 in the patient group treated with chemotherapy.

Abbreviations: ECOG PS, Eastern Cooperative Oncology Group Performance Status; NA, not available; WD, well-differentiated; MD, moderately differentiated; IALN, intra-abdominal lymph node.

## Discussion

We found that the factors independently prognostic of poor OS in patients with advanced SBA were absence of chemotherapy, no prior tumor resection, liver metastasis, and IALN metastasis. After adjustment of clinical factors using the IPTW method, chemotherapy status remained significantly associated with OS.

Of the four factors independently predictive of poor prognosis, three have previously been identified in patients with advanced SBA; these are absence of chemotherapy [7,8], no prior tumor resection [8-11], and liver metastasis [11]. The tumor burden of patients experiencing recurrence after curative resection, or metastasis after palliative resection has been regarded as lower than the burden in patients who did not undergo surgical resection. Although duodenal tumor location and peritoneal seeding have been reported to be predictive of poor prognosis in other series [7,10-12], they were not statistically significant in our analysis.

In agreement with previous retrospective studies, our findings suggest that palliative chemotherapy may benefit patients with SBA [7,9,13-16]. As patients who did not receive chemotherapy showed the poorest prognosis, we sought to estimate the effects of chemotherapy. In principle, the best way to evaluate the efficacy of a treatment is to employ a prospective randomized comparison study, but this is near-impossible because of the rarity of SBA. Therefore, the clinical backgrounds of patients in each group were adjusted using propensity scoring, to minimize selection bias, and the survival of patients in the chemotherapy and non-chemotherapy groups was compared. As we could not perform analyses using score-matched pairs, because of the small sample size, we utilized the IPTW method to show that chemotherapy could significantly improve OS in patients with advanced SBA.

The response rate (11%) and survival outcome (median OS, 11.8 months) observed in patients receiving chemotherapy were comparable to those previously reported [9,10,12,13,15-18]. However, an optimal chemotherapy regimen remains to be defined. Chemotherapy in patients with SBA is guided primarily by the treatment of patients with colorectal or upper gastrointestinal tract cancers. FOLFOX has been shown to enhance both OS and PFS, compared with LV5FU2-cisplatin [11]. A recent prospective Phase II trial of capecitabine-plus-oxaliplatin in patients with advanced SBA and ampullary adenocarcinoma yielded an overall RR of 50%, a median time-to-progression of 11.3 months, and a median OS of 20.4 months [19].

Our results should be interpreted with caution because this study had several limitations including its retrospective design and patients from a single institution, which could have selection biases. Bone and lung metastasis could be underestimated because bone scan and chest CT were not mandatory for the evaluation of disease extent. Furthermore, chemotherapy treatments were not homogeneous even though the majority of regimens consisted of fluoropyrimidine-based chemotherapy. Nonetheless, it should be considered that our cohort is one of the largest advanced SBA group evaluated to date.

## Conclusions

In conclusion, we identified four factors predictive of poor prognosis in patients with advanced small bowel adenocarcinoma. After IPTW adjustment, using propensity scoring to reduce selection bias, we found that patients who did not receive chemotherapy were at a significantly higher risk of mortality. Our findings indicate that chemotherapy may improve survival outcomes in patients with advanced SBA.

## Competing interests

The authors declare that they have no competing interests.

## Authors' contributions

DHK and TWK had full access to all of the data in the study and take responsibility for the integrity of the data and the accuracy of the data analysis. DHK, SCY, YSH and TWK were involved in study concept and design. MHR, JLL, HMC, BYR, YKK and TWK were involved in acquisition of data. DHK, SCY, YSH and TWK analyzed and interpreted the data. DHK and TWK drafted the manuscript. SCY, YSH MHR, JLL, HMC, BYR, YKK and TWK critically revised the manuscript for important intellectual content. DHK, SCY and TWK provided statistical expertise. TWK obtained funding. DHK, SCY and TWK supervised the study.

## Pre-publication history

The pre-publication history for this paper can be accessed here:

http://www.biomedcentral.com/1471-2407/11/205/prepub

## References

[B1] Jemal A Siegel R Xu J Ward E Cancer Statistics, 2010 CA: A Cancer Journal for Clinicians 2010 60 5 277 300 10.3322/caac.20073 20610543

[B2] Registry of Korea Central Cancer, Ministry for Health Welfare and Family Affairs Cancer Incidence in Korea 1999-2002 2008 http://www.cancer.go.kr/cms/data/edudata/__icsFiles/afieldfile/2009/09/16/aa%28aa0606%29.pdf [Accessed September 25, 2010]

[B3] Haselkorn T Whittemore AS Lilienfeld DE Incidence of Small Bowel Cancer in the United States and Worldwide: Geographic, Temporal, and Racial Differences Cancer Causes and Control 2005 16 7 781 787 10.1007/s10552-005-3635-6 16132788

[B4] Overman MJ Recent advances in the management of adenocarcinoma of the small intestine Gastrointest Cancer Res 2009 3 3 90 96 19626152 PMC2713134

[B5] D'Agostino RB Propensity score methods for bias reduction in the comparison of a treatment to a non-randomized control group Statistics in Medicine 1998 17 19 2265 2281 10.1002/(SICI)1097-0258(19981015)17:19<2265::AID-SIM918>3.0.CO;2-B 9802183

[B6] Robins JM Hernan MA Brumback B Marginal Structural Models and Causal Inference in Epidemiology Epidemiology 2000 11 5 550 560 10.1097/00001648-200009000-00011 10955408

[B7] Fishman PN Pond GR Moore MJ Oza A Burkes RL Siu LL Feld R Gallinger S Greig P Knox JJ Natural History and Chemotherapy Effectiveness for Advanced Adenocarcinoma of the Small Bowel: A Retrospective Review of 113 Cases American Journal of Clinical Oncology 2006 29 3 225 231 210.1097/1001. coc.0000214931.0000201062.000021490110.1097/01. coc.0000214931.01062.01 16755174

[B8] Dabaja BS Suki D Pro B Bonnen M Ajani J Adenocarcinoma of the small bowel: presentation, prognostic factors, and outcome of 217 patients Cancer 2004 101 3 518 526 10.1002/cncr.20404 15274064

[B9] Overman MJ Kopetz S Wen S Hoff PM Fogelman D Morris J Abbruzzese JL Ajani JA Wolff RA Chemotherapy with 5-fluorouracil and a platinum compound improves outcomes in metastatic small bowel adenocarcinoma Cancer 2008 113 8 2038 2045 10.1002/cncr.23822 18759326

[B10] Moon Y Rha S Shin S Chang H Shim H Roh J Adenocarcinoma of the small bowel at a single Korean institute: management and prognosticators Journal of Cancer Research and Clinical Oncology 2010 136 3 387 394 10.1007/s00432-009-0668-3 19760196 PMC11828314

[B11] Zaanan A Costes L Gauthier M Malka D Locher C Mitry E Tougeron D Lecomte T Gornet JM Sobhani I Chemotherapy of advanced small-bowel adenocarcinoma: a multicenter AGEO study Annals of Oncology 2010 21 9 1786 1793 10.1093/annonc/mdq038 20223786

[B12] Gibson MK Holcroft CA Kvols LK Haller D Phase II study of 5-fluorouracil, doxorubicin, and mitomycin C for metastatic small bowel adenocarcinoma Oncologist 2005 10 2 132 137 10.1634/theoncologist.10-2-132 15709215

[B13] Suenaga M Mizunuma N Chin K Matsusaka S Shinozaki E Oya M Ueno M Yamaguchi T Muto T Konishi F Hatake K Chemotherapy for small-bowel Adenocarcinoma at a single institution Surgery Today 2009 39 1 27 31 10.1007/s00595-008-3843-2 19132464

[B14] Czaykowski P Hui D Chemotherapy in small bowel adenocarcinoma: 10-year experience of the British Columbia Cancer Agency Clin Oncol (R Coll Radiol) 2007 19 2 143 149 10.1016/j.clon.2006.12.001 17355111

[B15] Locher C Malka D Boige V Lebray P Elias D Lasser P Ducreux M Combination Chemotherapy in Advanced Small Bowel Adenocarcinoma Oncology 2005 69 4 290 294 10.1159/000089678 16282708

[B16] Crawley C Ross P Norman A Hill A Cunningham D The Royal Marsden experience of a small bowel adenocarcinoma treated with protracted venous infusion 5-fluorouracil Br J Cancer 1998 78 4 508 510 10.1038/bjc.1998.523 9716035 PMC2063096

[B17] Ono M Shirao K Takashima A Morizane C Okita N Takahari D Hirashima Y Eguchi-Nakajima T Kato K Hamaguchi T Yamada Y Shimada Y Combination chemotherapy with cisplatin and irinotecan in patients with adenocarcinoma of the small intestine Gastric Cancer 2008 11 4 201 205 10.1007/s10120-008-0484-5 19132481

[B18] Hong SH Koh YH Rho SY Byun JH Oh ST Im KW Kim EK Chang SK Primary Adenocarcinoma of the Small Intestine: Presentation, Prognostic Factors and Clinical Outcome Jpn J Clin Oncol 2009 39 1 54 61 18997182 10.1093/jjco/hyn122

[B19] Overman MJ Varadhachary GR Kopetz S Adinin R Lin E Morris JS Eng C Abbruzzese JL Wolff RA Phase II Study of Capecitabine and Oxaliplatin for Advanced Adenocarcinoma of the Small Bowel and Ampulla of Vater J Clin Oncol 2009 27 16 2598 2603 10.1200/JCO.2008.19.7145 19164203

